# Responses of Jumbo Quail to a Diet Containing Corticated Marama Bean (*Tylosema esculentum*) Meal Pre-Treated with Fibrolytic Multi-Enzymes

**DOI:** 10.3390/life14101242

**Published:** 2024-09-28

**Authors:** Mveleli Marareni, Chidozie Freedom Egbu, Cornelia Kedidimetse Lebopa, Caven Mguvane Mnisi

**Affiliations:** 1 Department of Animal Science, Faculty of Natural and Agricultural Science, North-West University, P Bag x2046, Mmabatho 2735, South Africa; mvelelimarareni@gmail.com (M.M.); 33102643@mynwu.ac.za (C.F.E.); cornelia.lebopa@nwu.ac.za (C.K.L.); 2 Food Security and Safety Focus Area, Faculty of Natural and Agricultural Sciences, North-West University, P Bag x2046, Mmabatho 2735, South Africa

**Keywords:** dietary fibre, exogenous enzymes, growth performance, marama bean, meat quality, nutrient sources, quail

## Abstract

The nutritional utility of leguminous products such as corticated marama bean (*Tylosema esculentum*) meal (CMBM) in quail diets is limited by high fibre levels. This study evaluated the impact of dietary CMBM pre-treated with fibrolytic multi-enzyme (FMENZ) on growth performance, and physiological and meat quality responses in Jumbo *Coturnix* quail. Two hundred and forty 7-day-old Jumbo quail (29.4 ± 2.72 g initial live weight) were randomly distributed to five experimental diets, with six replicate cages each (eight birds/cage). The diets were a grower diet without CMBM, and the same grower diet plus 100 g/kg CMBM pre-treated with 0, 1, 1.5, and 2% (*v*/*w*) FMENZ. Positive quadratic responses (*p* < 0.05) were recorded for overall feed intake and body weight gain in weeks 2 and 3. The control diet promoted the highest (*p* < 0.05) gain-to-feed ratio in weeks 2 and 3, and the best weight gains and glucose levels, but reduced lipase levels. Final body weights declined linearly [*p* = 0.037] with FMENZ levels. Breast pH increased linearly, while haemoglobin and 1 h post-mortem chroma showed positive quadratic effects (*p* < 0.05) with FMENZ levels. The use of the enzymes did not improve the feed value of CMBM in Jumbo quail diets.

## 1. Introduction

Quail farming has gained significant attention globally because of their fast growth rates, quick returns on investments, high laying rate, and lower land requirements [1,2,3]. Jumbo quail are a larger variety of the common *Coturnix* quail, which were bred specifically for meat production, for their heavy body weights, and strong adaptability to various climates [4,5]. Unlike chickens, Jumbo quail have minimal environmental carbon footprints and require less rearing space, making them ideal for small-scale and sustainable farming operations in rural and urban areas [6]. In response to global food security challenges, diversification of the poultry industry with rapidly growing birds such as Jumbo quail is touted as a sustainable endeavour to expand the pool of animal protein sources [7,8]. Furthermore, quail farming presents an opportunity to create sustainable streams to supply animal protein even in low-income countries [9,10]. However, the cost and availability of major conventional grains that are largely used in quail diets pose sustainability challenges for quail farming globally. Marama bean (MB; *Tylosema esculentum*) is a legume native to southern Africa, where it is gaining research interest due to its high crude protein value (327 g/kg dry matter [DM]) [11]. Additionally, it has a high oil content that varies between 24 and 48% on a DM basis, which is predominantly composed of mono- and diunsaturated fatty acids and devoid of cholesterol [12,13]. Moreover, it contains essential nutrients such as calcium, phosphorus, potassium, sulphur, zinc, iron, vitamins B and E [12,13], and magnesium [14] that could satisfy the nutritional requirements of Jumbo *Coturnix* quail. Interestingly, it possesses biologically active phenolic substances such as gallic acid, ferulic acid, catechin, naringin, rutin, quercetin, and kaempferol that have antioxidant activities [13]. However, corticated MB contains high dietary fibre (190–270 g/kg DM) levels [12], which limits its utilisation efficiency in Jumbo quail [15].

Indeed, Jumbo quail reared on diets containing 150 g/kg olive pomace, a fibrous agro-waste by-product, had poor growth performance and carcass yield [16]. Similarly, dietary inclusion of canola meal at 300 g/kg reduced feed intake, egg weight, and feed conversion efficiency of Japanese quail [17]. This is attributable to the large proportion of undigested non-starch polysaccharides (NSPs), which account for a significant amount of digesta in high-fibre diets, resulting in digestive disturbances and stunted growth in young developing birds [18,19]. Furthermore, high fibre levels in poultry diets enable the production of high-molecular weight clinging lumps (i.e., beta-glucan) in the gastrointestinal system, which inhibit digesta flow [20]. This results in excess nutrient excretion, and poor feed efficiency and growth, all of which compromise the overall performance of Jumbo quail [15,16].

Decortication of MB could reduce the negative effects of fibre; however, this process is laborious, expensive, difficult to execute, and could be prohibitive in large-scale processing [13,15]. Thus, pre-treatment of corticated marama bean meal (CMBM) with exogenous fibrolytic enzymes could be an effective technique to negate the antinutritional effects of fibre for enhanced utilisation by Jumbo quail. Exogenous fibrolytic multi-enzymes (FMENZs) degrade the cell wall matrix of structural carbohydrates and improve nutrient utilisation and digestibility in monogastric animals [21,22]. Thus, pre-treatment of CMBM with exogenous FMENZ would aid in the digestion of NSPs in CMBM and improve its nutritional quality and subsequent utilisation by the birds. However, to the best of our knowledge, the application of FMENZ to enhance the utility of CMBM for the Jumbo quail has not been explored. Therefore, we assessed the impact of exogenous FMENZ pre-treatment of CMBM on growth performance, blood parameters, carcass and visceral morphometry, and meat quality traits in Jumbo quail. 

## 2. Materials and Methods

### 2.1. Marama Collection and Pre-Processing

Full-fat corticated MB (CMB) was hand-collected in Malwelwe rangeland (23.94282° S; 25.1999° E) in Botswana. The CMB was crushed and milled using a Trapp forage shredder (TFR 400, Jaraguá do Sul, Brazil) and then homogenised (2 mm) using a Polymix grinder (PX-MFC 90 D, Kinematica AG, Laufenburg, Switzerland). The processing of the beans is depicted in Plates S1, S2, S3, and S4. Thereafter, 10 kg samples of CMBM were each pre-treated with FMENZ (Viscozyme^®^ L, Sigma-Aldrich, Modderfontein, South Africa) at rates of 0, 1, 1.5, and 2% as described by Mulaudzi et al. [23]. This FMENZ cocktail consisted of cellulase, hemicellulase, xylanase, β-glucanase, and arabinose. The enzyme cocktail had an enzyme activity equivalent to 100 fungal β-glucanase per gram and a 1.2 g/mL density. After enzyme application, the samples were air-dried to constant weight and homogenised to blend with the other ingredients that were purchased from Nutroteq and SimpleGrow Agric Services (Centurion, South Africa).

### 2.2. Diet Formulation and Analyses

The diets (~1 mm mash) were formulated (Table 1) to meet the nutritional requirements of growing *Coturnix* quail [24] and were as follows: a grower quail diet without CMBM (CON); and CON plus 100 g/kg CMBM pre-treated with 0 (CMBZ0), 1 (CMBZ10), 1.5 (CMBZ15), and 2% (*v*/*w*) FMENZ (CMBZ20).

The CMBM samples (Table 2) and diets (Table 3) were analysed for dry matter (method no. 930.15), organic matter (method no. 942.05), and crude protein (method no. 984.13) following Association of Official Analytical Chemists [25] methods. The automated ANKOM^DELTA^ Fiber Analyzer (ANKOM Technologies, 2052 O’Neil Road Macedon, NY, USA) was used to assay for crude fibre, neutral detergent fibre (NDF), and acid detergent fibre (ADF) as described by Van Soest et al. [26]. The ADF residue bags were soaked in 72% sulphuric acid for 3 h to determine acid detergent lignin (ADL). Ether extract of MBM substrates was measured by extraction with petroleum ether for 120 min using an ANKOM^XT15^ Extractor (ANKOM Technologies, 2052 O’Neil Road Macedon, NY, USA). Essential amino acids (lysine, methionine, and threonine) were analysed at Stellenbosch University [27]. Agri-Laboratory Association of Southern Africa [28] guidelines were used to quantify selected minerals (calcium, chloride, phosphorus, and sodium). A bomb calorimeter (model no. MC-1000, Energy Instrumentation (Pty) Ltd., Knoppieslaagte, Centurion, South Africa) was used to determine gross energy at the Nutrilab at the University of Pretoria, South Africa.

### 2.3. Experimental Design and Growth Trial

The feeding trial took place at Molelwane Research Farm (25°86′00″ S; 25°64′32″ E) of the North-West University, South Africa. Two hundred and forty 7-day-old unsexed Jumbo quail, in a completely randomised design, were evenly allocated into 30 replicate cages (100 cm long × 60 cm wide × 30 cm high) considered as the experimental units. The five experimental diets were randomly distributed to generate 6 replicate cages holding 8 birds. Measurements were taken from 7 to 35 days of age, and the trial was carried out under natural daylight (approximately 12 h daylight intervals). Clean drinking water and experimental diets were provided ad libitum throughout the trial. The birds were reared under natural lighting from 06h00 to 18h00. The house was well ventilated by opening windows during the day to allow air flow and a relative humidity of 60%, with house temperature ranging from 27 to 33 °C.

### 2.4. Growth Performance Measurements

Initial body weights, averaging 29.42 ± 2.72 g, were weighed at 7 days of age and thereafter measured weekly per cage to calculate average weekly body weight gain. Daily feed intake was calculated using the quantity of feed supplied the previous day and the remaining feed the next morning and reported as the average weekly feed intake. The gain-to-feed ratio (G:F) was calculated by dividing body weight gain by the feed intake. During the study, the weights of the carcasses were recorded and used to correct the G:F.

### 2.5. Slaughter Technique, Blood Sampling, and Evaluation

All the birds in each replicate cage were weighed at 35 days of age to establish their final body weight. The birds were placed in well-ventilated crates per experimental unit and transported for 13 miles to a poultry slaughterhouse situated in Rooigrond (North West, South Africa). The birds were exsanguinated by slicing the jugular vein after two hours of resting. At least 4 mL of fresh blood was obtained randomly from two quail per experimental unit during bleeding. Whole blood tubes (with an anticoagulant) were kept in a cooler and the blood was examined within 48 h of collection. The automated LaserCyte Haematology Analyzer (model no. 933000101, IDEXX Laboratories (Pty) Ltd., Johannesburg, South Africa) was used to examine red blood cells (RBCs), red cell distribution width (RDW), neutrophil, lymphocytes, monocytes, eosinophil, haematocrit, mean corpuscular volume (MCV), mean cell haemoglobin concentration (MCHC), haemoglobin, and mean corpuscular haemoglobin (MCH). The blood samples in serum tubes were centrifuged at 2659.95 RCF (Cryste Varispin 4 Multi-purpose centrifuge, Cryste Co., Ltd, Seokcheon-ro, Bucheon-si, Gyeonggi-do, Republic of Korea) to generate serum. The serum biochemical indicators, viz., glucose, symmetric dimethylarginine (SDMA), phosphorus, calcium, total protein, globulin, albumin/globulin ratio (ALB/GLOB), alkaline phosphatase (ALKP), cholesterol, amylase, lipase, and alanine transaminase (ALT), were analysed using an automated Catalyst One Chemistry Analyzer (model no. 89-92525-00, IDEXX Laboratories (Pty) Ltd., Johannesburg, South Africa).

### 2.6. Carcass and Visceral Morphometry Measurements

The carcasses were weighed and eviscerated to determine hot carcass weight (HCW), then cooled for 24 h at 4 °C to measure cold carcass weight (CCW). Carcass characteristics such as breast, drumstick, wing, and thigh weights were measured using a weighing scale (Explorer^®^ EX224, OHAUS Corp, Parsippany, NJ, USA). The dressing percentage was calculated as the ratio of CCW to final body weight. The relative weights of the gizzard, liver, spleen, and proventriculus were recorded using the above-mentioned digital scale, while the lengths of the small and large intestines were estimated with a measuring tape (cm).

### 2.7. Meat Quality Evaluation

Breast meat pH was measured at 1 h and 24 h after slaughter using a digital meter (Corning Model 4, Corning Glass Works, Medfield, MA, USA) fitted with a spear-piercing electrode. For each replicate cage, standard solutions (pH 4, 7, and 10) were utilised to calibrate the meter. At 1 and 24 h post-mortem, breast meat colour coordinates, namely, lightness (L*), redness (a*), and yellowness (b*), were assessed using a colorimeter as prescribed by CIE [29]. The a* and b* values were used to calculate breast meat chroma and hue angle values [30]. Drip loss was assessed after hanging breast meat samples using wire steel and stored in a cold chamber (4 °C) for 72 h [31]. Cooking losses in breast meat were determined upon cooking the samples to a core temperature of 75 °C [32].

### 2.8. Statistical Analyses

Weekly feed intake, weight gain, and G:F were evaluated by applying repeated-measures analysis in the procedure of general linear model in SAS [33] to examine the interactive effects between feed and time. Dietary variations among parameters were evaluated by employing a one-way analysis of variance in SAS. Linear and quadratic coefficients for all measured parameters (except CON data) were assessed using the procedure of response surface regression in SAS [33] using the following quadratic equation:

$$y=ax^{2}+bx+c$$

where *y* was the response variable, *a* and *b* were the coefficients of the quadratic equation, *c* was the intercept, and *x* was the FMENZ level. Responses that minimised or maximised the parameters from the quadratic equation were calculated as *x* = −*b*/2*a*. The significance threshold was set at *p* < 0.05, and SAS’s probability of difference package was utilised to contrast the treatment means.

## 3. Results

### 3.1. Chemical Components

The non-treated substrate (MBZ0) had numerically lower crude protein (CP) and extract (EE) content which tended to increase with FMENZ levels (Table 2). Likewise, increasing FMENZ levels numerically reduced NDF and ADF values. However, the MBZ0 group had higher numerical values for lysine, methionine, and threonine than the FMENZ-treated groups.

Table 3 shows that CMBZ0 had a higher numerical crude protein value than the other treatments. The CON diet had the lowest crude fibre content among the others. Furthermore, the ether extract content of the diets showed a numerical variation in response to enzyme treatment. The FMENZ-treated group had better values for lysine, methionine, and threonine than the CMBZ0 group.

### 3.2. Growth Performance

There were significant week × treatment interaction effects on average body weight gain (*p* = 0.0001) and G:F (*p* = 0.0001) but not on feed intake (*p* = 0.280). Table 4 indicates that there was a positive quadratic and negative linear effect for overall feed intake [*y* = 0.09 (±0.036) *x*^2^ − 2.15 (±0.692) *x* + 108.2 (±2.602); R^2^ = 0.510; *p* = 0.027], which was minimised at 1.19% FMENZ level. Quail reared on CON had the highest (*p* < 0.05) overall feed intake, followed by those on CMBZ0, CMBZ15, and CMBZ20, while those on CMBZ10 exhibited the lowest feed intake. Increasing FMENZ levels induced positive quadratic effects for weight gain in week 2 [*y* = 0.09 (±0.028) *x*^2^ − 1.53 (±0.538) *x* + 20.59 (±2.023); R^2^ = 0.489; *p* = 0.007] and week 3 [*y* = 0.07 (±0.033) *x*^2^ − 1.61 (±0.638) *x* + 32.36 (±2.299); R^2^ = 0.367; *p* = 0.048]. The quadratic responses show that BWG in weeks 2 and 3 was minimised at 0.85 and 1.15% FMENZ levels, respectively. A positive quadratic response was also observed for G:F in week 2 [*y* = 0.001 (±0.0004) *x*^2^ − 0.02 (±0.008) *x* + 0.33 (±0.029); R^2^ = 0.416; *p* = 0.024], which was minimised at 1.0% FMENZ level.

There were no dietary effects on initial body weight and mortality (*p* > 0.05). Diet CON promoted the highest (*p* < 0.05) final body weight (FBW), followed by CMBZ0, and the lowest values were from CMBZ10 and CMBZ15. However, the CMBZ20 treatment was statistically similar (*p* > 0.05) to CMBZ0, CMBZ10, and CMBZ15 in terms of FBW. Two-week-old birds fed CON exhibited the highest (*p* < 0.05) weight gain (37.1 g/bird) whereas those on CMBZ10 (15.9 g/bird) had the lowest weight gain. In weeks 3 and 4, birds on CON had the highest weight gain and the lowest values were from those fed diets CMBZ10 and CMBZ15. Five-week-old quail on CMBZ20 had the lowest weight gain compared with the birds on the other treatments, which did not differ from each other (*p* > 0.05). Two-week-old birds on CON had the highest (*p* < 0.05) G:F and the lowest was from birds on CMBZ10. Three-week-old birds on CON had the highest (*p* < 0.05) G:F, followed by those on CMBZ0, CMBZ10, and CMBZ20, and the lowest G:F was from those on CMBZ15.

### 3.3. Blood Indices

Table 5 indicates that there were no linear or quadratic effects (*p* > 0.05) for blood parameters, apart from haemoglobin which showed a positive quadratic response effect [*y* = 0.009 (±0.004) *x*^2^ − 0.19 (±0.079) *x* + 12.63 (±0.296); R^2^ = 0.339; *p* = 0.039], which was minimised at 1.06% FMENZ level. Birds on CON had the highest (*p* < 0.05) glucose content followed by those on CMBZ0, CMBZ10, and CMBZ15, and the lowest was from CMBZ20. The CON group had the lowest lipase compared with the other treatment groups, which did not vary from each other (*p* > 0.05).

### 3.4. Carcass and Visceral Morphometry

Table 6 shows that slaughter weight [*y* = 161.8 − 3.06 (±1.608) *x*; R^2^ = 0.3746; *p* = 0.037] decreased linearly with FMENZ levels. Positive quadratic effects were observed for HCW [*y* = 0.19 (±0.081) *x*^2^ − 4.58 (±1.554) *x* + 109.7 (±5.848); R^2^ = 0.4885; *p* = 0.036] and CCW [*y* = 0.19 (±0.080) *x*^2^ − 4.44 (±1.538) *x* + 108.3 (±5.785); R^2^ = 0.480; *p* = 0.040], which were minimised at 1.2 and 1.17% FMENZ levels, respectively. However, a negative quadratic effect was noted for the relative weight of the gizzard [*y* = 0.19 (±0.066) *x* − 0.009 (±0.003) *x*^2^ + 2.86 (±0.248); R^2^ = 0.414; *p* = 0.015], which was maximised at 1.06%. Diet CON promoted the highest (*p* < 0.05) HCW and CCW, followed by CMBZ0, and the lowest values were from CMBZ10 and CMBZ15. However, the CMBZ20 treatment was comparable (*p* > 0.05) to CMBZ0, CMBZ10, and CMBZ15. The CON group had lower (*p* < 0.05) relative gizzard weights than all the other treatment groups.

### 3.5. Breast Meat Quality

Table 7 indicates that there was a linear increase in 1 h post-mortem pH (pH_1_) [*y* = 0.0003 (±0.001) *x* + 5.87 (±0.031); R^2^ = 0.1860; *p* = 0.026] and a positive quadratic trend for 1 h post-mortem breast meat chroma_1_ [*y* = 0.009 (±0.002) *x*^2^ − 0.136 (±0.043) *x* + 6.42 (±0.163); R^2^ = 0.2454; *p* = 0.002], which was minimised at 0.76%. However, negative quadratic effects were noted in 24 h post-mortem pH (pH_24_) [*y* = 0.003 (±0.001) *x* − 0.0001 (±0.0001) *x*^2^ + 6.07 (±0.036); R^2^ = 0.191; *p* = 0.029] and 1 h post-mortem redness (a*_1_) [*y* = 0.123 (±0.049) *x* − 0.007 (±0.003) *x*^2^ + 6.19 (±0.183); R^2^ = 0.416; *p* = 0.015], which were maximised at 1.5% and 0.88%, respectively. Birds on CMBZ20 had meat with a higher (*p* < 0.05) a*_1_ (7.709) than meat from the other treatments. Similarly, CMBZ20 promoted the (*p* < 0.05) highest meat chroma_1_ (15.24) while CMBZ0 (8.796) had the lowest. Birds fed diets CON and CMBZ20 had meat with lower (*p* < 0.05) pH_24_ values than those fed diets CMBZ0, CMBZ10, and CMBZ15.

## 4. Discussion

The application of exogenous NSP-degrading enzymes is recognised as an effective approach to enhance the utilisation of fibrous feed materials [34]. Nevertheless, no research has attempted to enhance the utility of CMBM in Jumbo quail diets using exogenous fibrolytic multi-enzymes (FMENZs). The use of FMENZ decreased fibre components in CMBM by breaking down the cell wall matrix of the substrate. This breakdown facilitates the accessibility of endogenous proteolytic and cellulolytic enzymes, aiding in the digestion of trapped proteins and carbohydrates [35]. Indeed, the FMENZ complexes increase protein content in CMBM by disintegrating the cell wall through hydrolysis of the linkages between structural polysaccharides, releasing intracellular proteins [36]. Regarding repeated-measures analysis, the significant diet and week interaction effects on BWG and G:F demonstrated that the ability of the birds to efficiently convert dietary treatments into body mass varied with age. With regards to the performance results, the CMBZ0 diet reduced overall FI more than the CON diet, which was anticipated given the high levels of fibre and NSP content in CMBM. These observations are consistent with the findings of Saki et al. [17], who reported a reduction in feed consumption in Japanese quail fed a 300 g/kg canola meal-containing diet compared to a soybean control diet. The positive quadratic effect on FI suggests that FMENZ did not enhance feed utilisation and nutrient availability. This implies that FMENZ levels beyond 1.19% did not facilitate fibre breakdown, leaving the substrate less digestible. Similarly, pre-treatment of CMBM with incremental levels of FMENZ resulted in positive quadratic effects for BWG (in weeks 2 and 3) and G:F (in week 2 only), further pointing to the inefficiency of the enzyme to improve the utility of CMBM for the Jumbo quail. Indeed, birds on CMBZ0 had higher average weekly weight gain than the enzyme-treated CMBM groups. Similarly, Mulaudzi et al. [23] reported no improvement in the growth performance of Jumbo quail after feeding them a diet containing moringa leaf powder pre-treated with 1% fibrolytic enzymes. Typically, NSP-rich plant cell walls act as barriers to nutrient absorption in monogastric animals such quail [18,19]. Furthermore, two-week-old birds fed CMBZ20 showed better G:F than those on CMBZ0 but this declined as the birds grew older. Contrary to our findings, an increase in weight gain and G:F was reported in broilers fed a canola meal and peas-based diet pre-treated with a mixture of carbohydrase enzymes at a rate of 0.1 g/kg [37] and 1.0 g keratinase/kg in a diet containing cottonseed meal [38]. Furthermore, an improvement in body weight gain and G:F in broilers fed a commercial maize soybean diet pre-treated with NSP multi-enzymes administered at rates of 250, 500, and 750 g/tonne was observed [39]. Mnisi et al. [40] also found higher FI with no improvements in BWG and G:F in female Japanese quail reared on diets containing canola pre-treated with 10% exogenous carbohydrases. The discrepancies among these studies could be attributed to the differences in the treated substrate type, application techniques, enzyme activities, and treatment doses [23].

Novel feed ingredients could affect the health status of the birds; hence, blood parameters are necessary to assess pathophysiological changes and the nutritional needs of quail [41]. The positive quadratic response for haemoglobin suggests that the treatments had negative alterations on blood pigments as FMENZ levels increased. As a critical component of red blood cells, haemoglobin is responsible for oxygen transport and distribution to tissues, which has a direct influence on total metabolic efficiency [42]. Thus, the observed results imply a threshold effect, in which the FMENZ treatments minimised the uptake of nutrients involved in haemoglobin synthesis such as iron and vitamin B_12_ [43], resulting in low haemoglobin concentration. These results disagree with those of Attia et al. [44], who reported an increase in the haemoglobin content of broilers fed a low-density diet treated with multi-enzymes at 0.2%. Furthermore, serum glucose and lipase levels are vital indicators of carbohydrate and lipid metabolism, respectively. The noted reduction in serum glucose and comparable lipase among the CMBM groups could have been influenced by the numerically higher ether extract values (Table 2 and Table 3). There is a metabolic interplay between fat and glucose metabolism. Hence, when fat digestion is enhanced, the body utilises more fatty acids for energy, potentially sparing glucose and leading to lower serum glucose levels [45]. The birds exhibited comparable total protein concentrations, indicating that the addition of FMENZ-treated CMBM had no compromising effect on the protein status of the birds [46]. Nonetheless, the blood parameters were within the normal reference levels stated for clinically healthy quail [47] except for the low levels of haematocrits, which did not differ from values reported for healthy quails. Furthermore, no dietary alterations in liver enzymes (ALT and ALKP) were identified, indicating that pre-treatment of CMBM with FMENZ did not impair the birds’ health.

Dressing percentage is critical for the economic viability of the poultry business [48]. Expectedly, CMBZ0 had lower carcass weights than the CON. Likewise, pre-treatment of CMBM with the enzyme did not improve carcass yields of the quail. This further suggests that the application of FMENZ did not improve the nutritional quality of CMBM, which is in line with the observed poor performance metrics. Our results agree with Mobeen et al. [49], who found no improvement in dressing percentage and carcass weights of Japanese quail reared on a diet containing 400 g/ton exogenous fibrolytic enzymes. However, Al-Flayyih and Kesab [50] observed improved dressing percentage and carcass weights when a soybean-based diet of Gambel and Bobwhite quail birds was supplemented with 0.03% multi-carbohydrase enzymes. The present findings also revealed a lack of treatment variation on all other carcass cuts, which is consistent with the findings of Mulaudzi et al. [23] in Jumbo quail reared on a diet containing moringa leaf powder pre-treated with 1% fibrolytic enzymes.

The relative weight of the gizzards increased, which was not expected since the application of FMENZ was intended to reduce the fibre content of CMBM. According to Moses et al. [51], feeding broiler chickens with fibrous diets induces visceral organ enlargement. Given that the gizzard relies on the structural components of feed to develop [52], its enlargement might be attributed to the inability of FMENZ to degrade the fibre content to the optimum threshold. Moreover, relative liver weights of quail reared on the FMENZ treatments were comparable in this study. Despite increases in serum lipase levels, it is possible that the influence on hepatic lipid accumulation was not sufficient to cause changes in liver weight. However, liver size is influenced by many factors other than lipid accumulation. The liver is a key location for lipid metabolism, and changes in this process can affect organ size [53].

Marama beans contain fats, fibre, amino acids, and minerals that may improve meat quality [11]. One of the most significant changes during rigour mortis is an alteration in pH, a critical factor that impacts meat colour, taste, and texture [54]. The pH of meat is influenced by the glycolytic pathway, where glycogen is converted to lactic acid post-mortem [55]. In this study, the comparable meat pH 1 h post-mortem suggests that the glycolytic pathway was relatively stable across all treatment groups. However, the increase in ultimate pH 24 h post-mortem during overnight storage in this study could have been a result of meat handling at various points during measurements. This increase primarily occurs due to the breakdown of amino acids and the subsequent production of ammonia through microbial processes [56]. Thus, the elevation in ultimate pH could not be attributed to a nutritional alteration; rather, it was associated with meat handling and storage. Meat colour is crucial because poultry consumers associate it with freshness, thus influencing their purchasing decisions. An L* range of 41.20–46.40 for quail meat is generally associated with freshness and high quality [57]. However, higher L* values indicate paleness and are often perceived as a sign of spoilage or poor quality [58]. In this study, CMBZ20 increased meat a*_1_ compared to other treatments, which is an effect that requires further research. Mir et al. [54] revealed that meat colour is dependent on myoglobin levels, with poor myoglobin oxygenation being related to high pH, resulting in dark meat. Since the meat pH (5.863–6.187) was within the typical range for fresh quail meat [16], the enhanced redness in this investigation might be related to greater oxygenation of myoglobin. Nonetheless, the absence of dietary effects on meat yellowness, lightness, hue angle, drip loss, cooking loss, and shear force indicates that the treatments did not alter the texture, appearance, and stability of Jumbo quail meat. 

## 5. Conclusions

It was concluded that pre-treatment of corticated marama bean meal with up to 2% fibrolytic multi-enzyme did not improve its feeding value for Jumbo quail. However, the results showed that overall feed intake and body weight gain declined linearly in response to enzyme treatment. Future studies should evaluate the use and cost-effectiveness of decorticated marama bean in diets of simple non-ruminants.

## Figures and Tables

**Table 1:** Total ingredient composition (g/kg as fed basis) of the diets.

	^1^ Diets	
Ingredients	CON	MBMd
Corticated marama bean meal	0.000	99.98
Yellow maize	552.2	584.5
Soya oil cake (46%)	266.9	244.73
Full fat soya	120.0	28.78
Limestone powder	12.44	12.00
Monocalcium phosphate	8.980	9.960
DL-Methionine	4.140	4.420
Fine salt	2.710	2.480
Sodium bicarbonate	1.270	1.630
Lysine-HCl	2.850	3.820
L-Threonine	1.120	1.300
L-Tryptophan	0.090	0.050
Crude soya oil mixer	20.95	0.000
^2^ Premix	3.000	3.000
^3^ Lignobond	2.000	2.000
Axtraphytase (10,000 FTU/g)	0.100	0.100
Salinomycin (12%)	0.500	0.500
Zinc bacitracin	0.500	0.500
Antioxidant	0.250	0.250
Total volume	1000	1000

^1^ Diets: CON = a grower diet without corticated marama bean meal; and MBMd = a grower diet with corticated marama bean meal. ^2^ Premix: vitamin A (11,000 IU), vitamin B_1_ (2.5 mg), vitamin B_2_ (4.5 mg), vitamin B_6_ (5.1 mg), vitamin D_3_ (2500 IU), vitamin E (25 IU), vitamin K_3_ (2.0 mg), biotin (0.12 g), folic acid (0.7 mg), niacin (30 mg), and pantothenic acid (10 mg). ^3^ Lignobond = high-performance lignin-based pellet binders.

**Table 2:** Analysed proximate and amino acid composition of corticated marama bean meal substrates with or without enzyme pre-treatment.

	^1^ Substrates			
Parameters	MBZ0	MBZ10	MBZ15	MBZ20
Gross energy (MJ/kg)	22.98	22.48	22.55	23.99
Dry matter (% DM)	92.91	93.94	92.51	93.21
Crude protein (% DM)	24.56	25.43	26.22	25.06
Ether extract (g/kg DM)	24.70	28.84	25.65	28.39
Neutral detergent fibre (% DM)	5.010	2.999	2.509	2.331
Acid detergent fibre (% DM)	4.760	1.644	1.514	1.167
Lysine (g/100 g DM)	2.110	1.110	0.980	1.430
Methionine (g/100 g DM)	0.390	0.230	0.150	0.200
Threonine (g/100 g DM)	1.440	0.990	0.710	0.930

^1^ Substrates: MBZ0 = corticated marama bean meal without enzymes; MBZ10 = corticated marama bean meal pre-treated with 1% fibrolytic enzymes; MBZ15 = corticated marama bean meal pre-treated with 1.5% fibrolytic enzymes; and MBM20 = corticated marama bean meal pre-treated with 2% fibrolytic enzymes.

**Table 3:** Analysed proximate and amino acid composition of the experimental diets.

	^1^ Diets				
Parameters	CON	CMBZ0	CMBZ10	CMBZ15	CMBZ20
Gross energy (MJ/kg)	16.80	17.50	17.50	17.84	17.83
Dry matter (%)	85.83	85.81	86.60	84.86	86.75
Crude protein (% DM)	22.08	21.32	22.61	21.97	22.35
Ether extract (% DM)	5.050	6.410	6.370	5.550	5.640
Neutral detergent fibre (g/kg DM)	9.619	10.43	10.26	9.736	9.626
Acid detergent fibre (g/kg DM)	3.468	4.330	4.063	3.705	3.857
Lysine (g/100 g DM)	1.010	1.170	1.180	1.150	1.270
Methionine (g/100 g DM)	1.080	0.750	1.050	1.360	0.930
Threonine (g/100 g DM)	1.070	0.810	0.810	0.880	0.830

^1^ Treatments: CON = a grower diet without corticated marama bean meal; CMBZ0 = a grower diet containing 100 g/kg non-treated corticated marama bean meal; CMBZ10 = a grower diet containing 100 g/kg corticated marama bean meal pre-treated with 1% fibrolytic enzymes; CMBZ15 = a grower diet containing 100 g/kg corticated marama bean meal pre-treated with 1.5% fibrolytic enzymes; and CMBZ20 = a grower diet containing 100 g/kg corticated marama bean meal pre-treated with 2% fibrolytic enzymes.

**Table 4:** Effects of pre-treating corticated marama bean meal with fibrolytic multi-enzymes on growth performance in Jumbo quail.

	^1^ Treatments						Significance		
Parameters	CON	CMBZ0	CMBZ10	CMBZ15	CMBZ20	SEM	P-_Overall_	P-_Linear_	P-_Quadratic_
Overall feed intake (g/bird)	123 ^a^	111 ^b^	97.2 ^c^	107 ^b^	105 ^b^	5.06	0.024	0.029	0.027
	Average body weight gain (g/bird)								
Week 2	37.10 ^a^	20.90 ^c^	15.90 ^d^	20.30 ^c^	25.30 ^b^	1.92	0.0001	0.353	0.007
Week 3	49.20 ^a^	32.40 ^b^	26.90 ^c^	25.30 ^c^	35.10 ^b^	2.30	0.0001	0.170	0.048
Week 4	42.50 ^a^	37.30 ^b^	32.20 ^c^	32.40 ^c^	36.70 ^b^	2.10	0.012	0.185	0.303
Week 5	39.44 ^a^	40.43 ^a^	37.16 ^a^	41.49 ^a^	26.46 ^b^	3.12	0.026	0.123	0.147
	Average gain-to-feed ratio								
Week 2	0.507 ^a^	0.337 ^c^	0.278 ^d^	0.343 ^c^	0.402 ^b^	0.029	0.0002	0.199	0.024
Week 3	0.442 ^a^	0.352 ^b^	0.329 ^b^	0.261 ^c^	0.361 ^b^	0.018	0.0001	0.172	0.249
Week 4	0.315	0.308	0.302	0.278	0.307	0.019	0.682	0.652	0.956
Week 5	0.229	0.246	0.262	0.272	0.189	0.020	0.092	0.338	0.056
Initial body weight (g/bird)	29.74	30.64	28.98	28.75	28.97	1.177	0.780	0.751	0.362
Final body weight (g/bird)	198.0 ^a^	161.7 ^b^	141.2 ^c^	148.3 ^c^	152.9 ^b,c^	6.219	0.0001	0.037	0.219
Mortality (%)	1.000	1.667	1.500	1.333	2.000	0.504	0.7036	0.816	0.463

^a,b,c,d^ In a row, values with varying superscripts are significantly different (*p* < 0.05). ^1^ Treatments: CON = a grower diet without corticated marama bean meal; CMBZ0 = a grower diet containing 100 g/kg non-treated corticated marama bean meal; CMBZ10 = a grower diet containing 100 g/kg corticated marama bean meal pre-treated with 1% fibrolytic enzymes; CMBZ15 = a grower diet containing 100 g/kg corticated marama bean meal pre-treated with 1.5% fibrolytic enzymes; and CMBZ20 = a grower diet containing 100 g/kg corticated marama bean meal pre-treated with 2% fibrolytic enzymes.

**Table 5:** Effects of pre-treating corticated marama bean meal with fibrolytic multi-enzymes on blood indices in Jumbo quail.

	^1^ Treatments						Significance		
^2^ Indices	CON	CMBZ0	CMBZ10	CMBZ15	CMBZ20	SEM	P-_Overall_	P-_Linear_	P-_Quadratic_
Haematological indices									
RBC (×10^12^/L)	1.889	2.393	1.826	1.741	2.220	0.300	0.513	0.506	0.082
RDW (×10^9^/L)	2.050	1.267	1.642	2.125	3.340	0.984	0.693	0.481	0.892
Neutrophil (×10^9^/L)	2.599	2.443	3.809	2.910	2.962	0.527	0.423	0.692	0.982
Lymphocyte (×10^9^/L)	0.338	0.281	0.248	0.206	0.413	0.105	0.708	0.883	0.120
Monocyte (×10^9^/L)	0.383	0.243	0.248	0.228	0.224	0.091	0.708	0.492	0.936
Eosinophil (×10^9^/L)	0.042	0.050	0.111	0.063	2.170	0.637	0.223	0.974	0.252
Haematocrits (%)	19.01	20.24	20.77	16.62	18.97	1.946	0.552	0.633	0.994
MCV	88.22	77.31	85.46	91.44	68.50	9.047	0.374	0.233	0.428
MCHC (%)	17.47	19.33	19.91	19.99	17.23	1.984	0.754	0.184	0.740
Haemoglobin (g/dL)	11.02	12.78	12.01	11.86	12.40	0.733	0.542	0.384	0.040
MCH (pg)	58.34	55.73	50.23	58.48	59.43	8.163	0.932	0.855	0.940
Serum biochemical indices									
Glucose (mmol/L)	10.69 ^a^	9.275 ^b^	8.954 ^b^	8.781 ^b^	7.426 ^c^	0.603	0.023	0.132	0.681
SDMA (μg/dL)	19.33	19.50	20.58	19.58	17.50	0.982	0.355	0.204	0.333
Phosphorus (mmol/L)	4.119	4.558	8.679	4.868	4.475	1.830	0.401	0.973	0.154
Calcium (mmol/L)	2.699	2.684	2.714	2.603	2.649	0.180	0.993	0.117	0.349
Total protein (g/L)	37.08	38.25	45.75	39.67	40.00	2.041	0.056	0.814	0.222
Globulin (g/L)	23.08	24.17	28.25	24.92	25.40	1.428	0.156	0.981	0.226
ALB/GLOB	0.608	0.592	0.608	0.600	0.590	0.025	0.977	0.441	0.278
ALKP (U/L)	341.8	297.8	334.0	294.8	460.0	65.08	0.460	0.828	0.499
Cholesterol (mmol/L)	4.480	4.082	4.265	4.176	4.683	0.280	0.609	0.328	0.635
Amylase (U/L)	277.8	354.5	527.9	349.0	430.9	62.78	0.091	0.938	0.622
Lipase (U/L)	245.8 ^b^	774.1 ^a^	670.7 ^a^	605.9 ^a^	605.1 ^a^	107.0	0.023	0.325	0.568
ALT (U/L)	29.60	40.83	63.83	49.90	40.51	9.573	0.133	0.188	0.252

^a,b,c^ In a row, values with varying superscripts are significantly different (*p* < 0.05). ^1^ Treatments: CON = a grower diet without corticated marama bean meal; CMBZ0 = a grower diet containing 100 g/kg non-treated corticated marama bean meal; CMBZ10 = a grower diet containing 100 g/kg corticated marama bean meal pre-treated with 1% fibrolytic enzymes; CMBZ15 = a grower diet containing 100 g/kg corticated marama bean meal pre-treated with 1.5% fibrolytic enzymes; and CMBZ20 = a grower diet containing 100 g/kg corticated marama bean meal pre-treated with 2% fibrolytic enzymes. ^2^ Indices: RBC = red blood cell; RDW = red blood cell distribution width; WBC = white blood cells; MCV = mean corpuscular volume; MCHC = mean corpuscular haemoglobin concentration; MCH = mean corpuscular haemoglobin; ALB/GLOB = albumin to globulin ratio; ALKP = alkaline phosphatase; ALT = alanine transaminase; and SDMA = symmetric dimethylarginine.

**Table 6:** Effects of pre-treating corticated marama bean meal with fibrolytic multi-enzymes on carcass and visceral morphometry (g/100 g CCW, except specified differently) in Jumbo quail.

	^1^ Treatments						Significance		
^2^ Parameters	CON	CMBZ0	CMBZ10	CMBZ15	CMBZ20	SEM	P-_Overall_	P-_Linear_	P-_Quadratic_
Dressing (%)	67.27	65.52	63.09	60.78	65.27	2.836	0.552	0.376	0.118
HCW (g)	133.0 ^a^	105.7 ^b^	89.28 ^c^	90.26 ^c^	99.77 ^b,c^	5.725	0.0001	0.032	0.036
CCW (g)	130.1 ^a^	104.2 ^b^	88.18 ^c^	88.32 ^c^	99.29 ^b,c^	5.678	0.0001	0.034	0.040
Breast	13.37	16.05	20.95	18.21	13.59	1.227	0.116	0.489	0.166
Drumstick	3.448	4.526	5.795	5.183	5.876	0.477	0.344	0.914	0.341
Wing	3.707	5.134	6.606	6.054	5.343	0.274	0.192	0.656	0.520
Thigh	5.321	6.743	8.760	8.028	8.633	0.561	0.278	0.913	0.609
Gizzard	1.769 ^b^	2.798 ^a^	3.838 ^a^	3.846 ^a^	2.831 ^a^	0.206	0.005	0.555	0.015
Liver	2.316	3.094	4.223	4.483	3.080	0.302	0.142	0.483	0.164
Spleen	0.079	0.107	0.147	0.143	0.106	0.014	0.540	0.657	0.185
Proventriculus	0.464	0.818	0.857	0.883	0.636	0.100	0.406	0.433	0.689
Small intestine (cm)	60.72	62.76	62.65	63.08	86.64	9.935	0.406	0.396	0.621
Large intestine (cm)	10.21	10.49	10.53	9.758	9.850	0.382	0.500	0.193	0.232

^a,b,c^ In a row, values with varying superscripts are significantly different (*p* < 0.05). ^1^ Treatments: CON = a grower diet without corticated marama bean meal; CMBZ0 = a grower diet containing 100 g/kg non-treated corticated marama bean meal; CMBZ10 = a grower diet containing 100 g/kg corticated marama bean meal pre-treated with 1% fibrolytic enzymes; CMBZ15 = a grower diet containing 100 g/kg corticated marama bean meal pre-treated with 1.5% fibrolytic enzymes; and CMBZ20 = a grower diet containing 100 g/kg corticated marama bean meal pre-treated with 2% fibrolytic enzymes. ^2^ Parameters: HCW = hot carcass weight; and CCW = cold carcass weight.

**Table 7:** Effects of pre-treating corticated marama bean meal with fibrolytic multi-enzymes on breast meat quality attributes in Jumbo quail.

	^1^ Treatment						Significance		
^2^ Parameters	CON	CMBZ0	CMBZ10	CMBZ15	CMBZ20	SEM	P-_Overall_	P-_Linear_	P-_Quadratic_
pH_1_	5.877	5.863	5.939	5.976	5.980	0.047	0.273	0.026	0.779
L*_1_ (lightness)	47.71	45.25	45.67	44.56	46.51	0.980	0.229	0.929	0.461
a*_1_ (redness)	6.589 ^b^	6.269 ^b^	5.834 ^b^	6.044 ^b^	7.709 ^a^	0.402	0.039	0.502	0.015
b*_1_ (yellowness)	2.470	1.519	1.913	1.911	2.699	0.277	0.051	0.084	0.343
Hue angle_1_	0.366	0.242	0.316	0.305	0.391	0.047	0.230	0.286	0.465
Chroma_1_	13.28 ^b^	8.796 ^e^	9.805 ^d^	9.949 ^c^	15.24 ^a^	1.257	0.006	0.065	0.002
pH_24_	5.993 ^b^	6.144 ^a^	6.187 ^a^	6.177 ^a^	6.058 ^b^	0.047	0.029	0.489	0.029
L*_24_ (lightness)	42.31	41.62	41.57	40.45	36.28	2.893	0.659	0.640	0.188
a*_24_ (redness)	8.518	7.361	7.179	7.496	29.13	8.611	0.389	0.193	0.171
b*_24_ (yellowness)	2.357	2.516	2.329	2.221	2.601	0.334	0.939	0.779	0.261
Hue angle_24_	0.272	0.330	0.314	0.287	0.393	0.033	0.132	0.137	0.361
Chroma_24_	14.34	13.93	12.86	12.87	16.67	1.352	0.285	0.409	0.129
Drip loss (%)	11.43	11.91	12.62	11.01	15.31	1.420	0.311	0.531	0.643
Cooking loss (%)	30.36	31.45	33.55	34.14	30.62	2.637	0.797	0.309	0.973

^a,b,c,d,e^ In a row, means with different superscripts vary (*p* < 0.05). ^1^ Treatments: CON = a grower diet without corticated marama bean meal; CMBZ0 = a grower diet containing 100 g/kg non-treated corticated marama bean meal; CMBZ10 = a grower diet containing 100 g/kg corticated marama bean meal pre-treated with 1% fibrolytic enzymes; CMBZ15 = a grower diet containing 100 g/kg corticated marama bean meal pre-treated with 1.5% fibrolytic enzymes; and CMBZ20 = a grower diet containing 100 g/kg corticated marama bean meal pre-treated with 2% fibrolytic enzymes. ^2^ Parameters: pH_1_ = pH 1 h post-mortem; and pH_24_ = pH 24 h post-mortem.

## Data Availability

All data supporting the findings of this study are available within the paper. The raw datasets used and analysed during the current study are available from the corresponding author upon reasonable request.

## Supplementary Materials

The following supporting information can be downloaded at: https://www.mdpi.com/article/10.3390/life14101242/s1.
