# Supplementary material for: Responses of Jumbo Quail to a Diet Containing Corticated Marama Bean (Tylosema esculentum) Meal Pre-Treated with Fibrolytic Multi-Enzymes
Source: Life (Basel). 2024 Sep 28;14(10):1242. doi: 10.3390/life14101242 (PMC11508706; doi:10.3390/life14101242)
Supplement: Supplementary file 1 [file life-14-01242-s001.zip › life-3207679-supplementary.pdf]

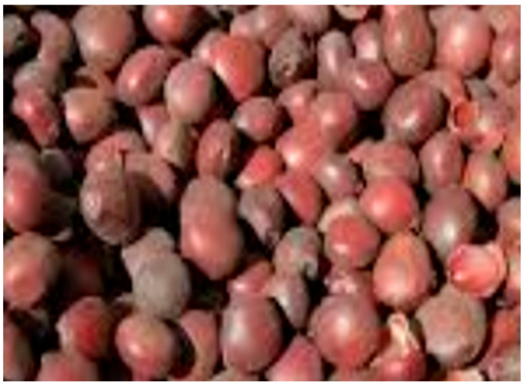
Plate S1: Whole Marama beans

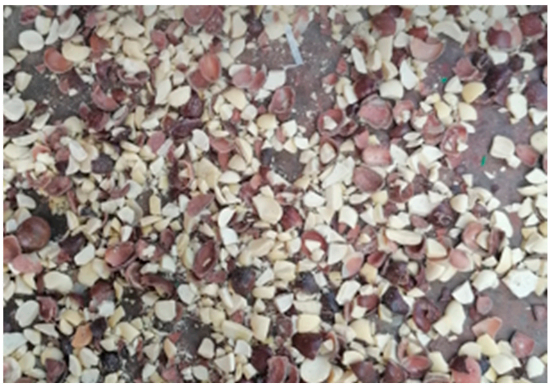
Plate S2: Crushed corticated Marama beans

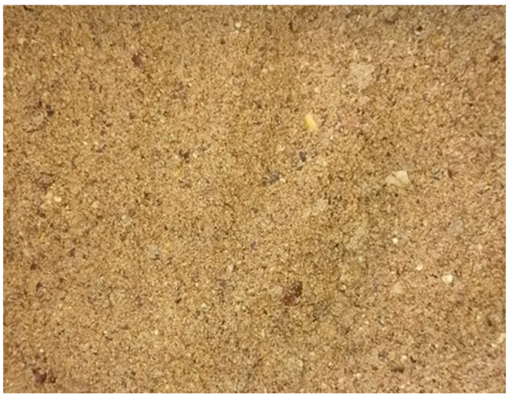
Plate S3: Milled corticated Marama beans

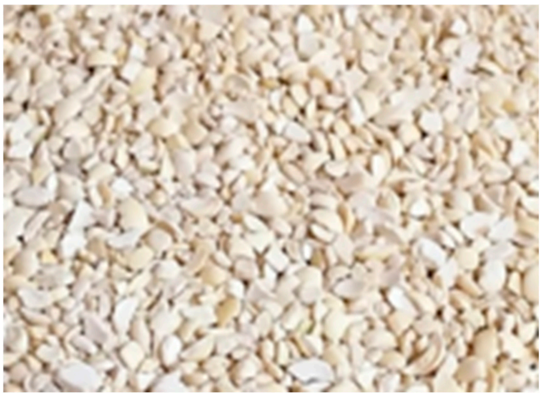
Plate S4: Crushed decorticated Marama beans
